# The Impact of Physician Advice on Lifestyle Changes Among Hypertensive Older Adults in The U.S.

**DOI:** 10.1093/geroni/igaf122.2386

**Published:** 2025-12-31

**Authors:** Joseph Phillips, Jun Chu

**Affiliations:** University of Maryland, Baltimore County, Baltimore, Maryland, United States; University of Maryland, Baltimore County, Baltimore, Maryland, United States

## Abstract

Hypertension affects half of the U.S. population and increases the risk of heart attacks, strokes, and kidney failure. Managing the progression of hypertension requires medication adherence and lifestyle changes, yet little is known about the prevalence of such changes after receiving medical advice from a physician. Additionally, variations in adherence across racial/ethnic groups remain unclear. Our objective was to measure the changes in medication intake and four other lifestyle measures (reducing alcohol consumption, reducing salt intake, eating healthier, and increasing exercise) among older hypertensive patients in the U.S. using the 2017 Behavioral Risk Factor Surveillance System’s Hypertension Awareness module. We estimated unadjusted and adjusted percentage point changes in groups with and without a physician’s advice using linear probability models, controlling for predisposing, enabling, and need factors. Our findings indicate that physician advice significantly increased rates of all five lifestyle changes across all tested racial/ethnic groups. Non-Latino Black patients exhibited the highest overall adherence, with or without advice. Among non-Latino White patients, the largest increases were in changing eating habits (27.02 percentage points), reducing salt intake (25.06 percentage points), reducing alcohol consumption (27.63 percentage points), and increasing exercise (22.21 percentage points). Non-Latino Black patients experienced notable increases in changing eating habits (20.58 percentage points) and increasing exercise (16.79 percentage points). This study demonstrates the importance of physician advice in managing hypertension changes among older adults, finding they are more likely to make lifestyle changes after receiving direct advice from medical providers.

